# The effect of fingerprint expertise on visual short-term memory

**DOI:** 10.1186/s41235-024-00539-9

**Published:** 2024-03-19

**Authors:** Brooklyn J. Corbett, Jason M. Tangen, Rachel A. Searston, Matthew B. Thompson

**Affiliations:** 1 School of Psychology, The University of Queensland, St Lucia, QLD 4072 Australia; 2 School of Psychology, The University of Adelaide, Adelaide, SA 5005 Australia; 3 School of Psychology, Murdoch University, Perth, WA 6150 Australia; 4 Centre for Biosecurity and One Health, Harry Butler Institute, Murdoch University, Perth, WA 6150 Australia

## Abstract

Expert fingerprint examiners demonstrate impressive feats of memory that may support their accuracy when making high-stakes identification decisions. Understanding the interplay between expertise and memory is therefore critical. Across two experiments, we tested fingerprint examiners and novices on their visual short-term memory for fingerprints. In Experiment 1, experts showed substantially higher memory performance compared to novices for fingerprints from their domain of expertise. In Experiment 2, we manipulated print distinctiveness and found that while both groups benefited from distinctive prints, experts still outperformed novices. This indicates that beyond stimulus qualities, expertise itself enhances short-term memory, likely through more effective organisational processing and sensitivity to meaningful patterns. Taken together, these findings shed light on the cognitive mechanisms that may explain fingerprint examiners’ superior memory performance within their domain of expertise. They further suggest that training to improve memory for diverse fingerprints could practically boost examiner performance. Given the high-stakes nature of forensic identification, characterising psychological processes like memory that potentially contribute to examiner accuracy has important theoretical and practical implications.

## Background

Our short-term memory allows us to briefly store and retrieve information to support ongoing cognition and task performance. Everyday activities like navigating a new driving route rely on short-term retention of key details. Likewise, specialised domains that demand real-time processing, such as sports, music, and medicine, depend heavily on short-term memory. For example, soccer players use their short-term memory to anticipate and respond to movements of their opponents and the ball (Ward & Williams, 2003). Musicians employ short-term memory to recall notes and phrases to compose impromptu pieces (Lehmann & Ericsson, 1993; Meinz & Hambrick, 2010). Experienced physicians analyse patient information held briefly in short-term memory to make diagnoses and treatment plans (Lesgold et al., 1988; Schmidt & Rikers, 2007). In each case, short-term retention of relevant details is essential for skilled performance and decision-making.

### Fingerprint examination

Experts in various fields likely rely on their memory to make decisions and solve problems within their domain. Understanding how expertise relates to short-term memory is particularly important in high-stakes domains like fingerprint examination where experts are required to make critical decisions. Fingerprint examination plays a critical role in forensic identification, relying on expert human examiners to analyse and compare ridge patterns across prints. Fingerprints have served as identification evidence for over a century (Cole, 2001), yet the subjective nature of comparisons has led scientific bodies to call for more research on the validity and reliability of fingerprint analysis (National Academy of Sciences, 2009; President's Council of Advisors on Science and Technology, 2016).

Though technology has increased automation, human examiners remain essential. They spend hours visually comparing prints from crime scenes to potential matches, determining if they come from the same source (Ashbaugh, 1999; Daluz, 2018; Ulery et al., 2011). Misidentifications can have serious consequences, like wrongful convictions, so accuracy is vital (Campbell, 2011; Thompson & Cole, 2005). Fingerprint examiners consistently outperform novices, even under constraints (Searston & Tangen, 2017; Tangen et al., 2011; Thompson & Tangen, 2014; Vogelsang et al., 2017). Yet the psychological processes underlying their expertise require further examination.

One potential factor contributing to examiners’ outstanding performance is short-term memory capacity. Their expertise may enhance retention for domain-relevant images like fingerprints, allowing more efficient encoding and comparison of prints (Ericsson & Kintsch, 1995; Gobet & Simon, 1996a). Quantifying expert-novice memory differences could therefore provide insight into the cognitive mechanisms supporting examiners’ accuracy.

### Short-term memory

Short-term memory has a limited capacity. Typically, only about 7 pieces or “chunks” of information can be retained, as suggested by Miller’s (1956) pioneering work. This capacity limit persists whether remembering digits, letters, nonsense syllables, or other materials. However, the amount of information held in short-term memory can vary depending on factors like the complexity of the information and a person’s expertise (Baddeley, 2000; Cowan, 2001; Ericsson & Kintsch, 1995; Gobet & Simon, 1996a, 1996b; Zhang & Luck, 2008).

For visual short-term memory specifically, capacity is constrained to about 3–4 objects (Luck & Vogel, 1997). The “slot” model proposes our memory system has a fixed number of slots, each storing a single object (Vogel et al., 2001). However, research shows visual short-term memory limitations also depend on an item’s complexity, with simpler objects (e.g. circles) remembered better than more complex, detailed ones (Alvarez & Cavanagh, 2004; Wheeler & Treisman, 2002). Retaining intricate visual details requires greater cognitive resources, further constraining memory capacity. In summary, short-term memory capacity is restricted but can vary based on factors like stimulus type and complexity which impact how efficiently information is processed and retained.

### Expertise and short-term memory

In addition to the type and complexity of information, a person’s level of expertise and experience within a domain impacts short-term memory performance. Numerous studies have demonstrated that experts exhibit superior retention for information related to their area of expertise compared to non-experts (Ericsson, 2018). For example, chess experts can recall the positions of pieces on a chessboard after brief exposures with much greater accuracy than novices (Chase & Simon, 1973; de Groot, 1965). Similarly, car experts have exhibited enhanced visual short-term memory specifically for cars compared to non-car experts (Curby et al., 2009).

In many other domains such as music (Lehmann & Ericsson, 1993; Meinz & Hambrick, 2010), sports (Ward & Williams, 2003), and medicine (Lesgold et al., 1988; Schmidt & Rikers, 2007), experts demonstrate similar patterns; they can process and retain information pertinent to their specific fields with greater accuracy and efficiency. Critically, these memory advantages do not extend beyond the domain of expertise, suggesting that they reflect an increased capacity for domain-specific information rather than a boost in general short-term memory skills (Ericsson, 2018).

Such enhancements in memory are not merely about storing more information; they involve complex processes like pattern recognition, predictive processing, and efficient information retrieval, which are crucial for performance in these fields (Ericsson, 2018). In sports, an athlete’s memory of past plays, opponents’ behaviours, and strategic knowledge contributes to their ability to anticipate and react effectively during a game (Ward & Williams, 2003). Similarly, a physician’s diagnostic acumen is largely dependent on their ability to recall and recognise patterns in symptoms and medical histories (Lesgold et al., 1988; Schmidt & Rikers, 2007). Understanding the role of memory in expertise provides valuable insights into how experts develop and maintain their skills.

The specific mechanisms underlying expertise effects on memory are not yet fully characterised, but several theories have been proposed. A key idea across explanations is that domain knowledge enhances organisational processing to more efficiently structure information. For instance, long-term working memory theory proposes that experts develop specialised long-term memory representations to store domain knowledge, freeing up limited short-term memory resources (Ericsson & Kintsch, 1995). Similarly, template theory suggests experts form abstract mental templates representing familiar patterns in their field, reducing short-term memory demands by matching new information to existing templates (Gobet & Simon, 1996a, 1996b). In fingerprint examination, templates could facilitate rapid identification and analysis of distinctive ridge patterns and minutiae. Overall, by leveraging their knowledge to organise information, experts can better manage short-term memory limits.

Some argue that enhanced expert memory relies on more than just organisational processing—item-specific processing also plays a role. The distinctiveness theory proposes that domain knowledge promotes memory by supporting effective processing of distinguishing details (Rawson & Van Overschelde, 2008). This involves identifying similarities between items as well as unique features of a particular item that differentiate it from related items (Hunt, 2003). For instance, bird experts remember species based on shared traits and taxonomic categories, but also by focusing on distinctive diagnostic features, like beak shape or plumage patterns, that distinguish similar species (Peeck & Zwarts, 1983). By noting both shared and distinctive attributes, experts can build rich, informative memory representations.

Like bird experts, fingerprint examiners must focus on subtle distinguishing details when comparing similar prints. This can be an extremely challenging perceptual task, as prints vary greatly in quality and distortion. Matching prints may look quite dissimilar due to differences in pressure, movement, recording methods, and environmental factors. On the other hand, non-matching prints can look very similar, particularly because of the increased reliance on computer algorithms which search huge databases and return lists of highly similar candidate prints for examiners to compare (Dror & Mnookin, 2010). Due to this complexity, experts must become accustomed to the many ways prints can vary between and within individuals. By integrating knowledge of typical patterns and distinguishing minutiae, fingerprint expertise likely supports enhanced memory capacity, much like bird expertise facilitates remembering species’ unique traits (Rawson & Van Overschelde, 2008).

To develop organised knowledge structures containing specific item details, experts may rely on analytic processing strategies (Towler et al., 2017; White et al., 2015b). Analytic processing involves systematically analysing information to identify defining features or categories that differentiate items. For example, fingerprint examiners carefully study minutiae patterns, learning to categorise details such as a short ridge that runs between two parallel ridges, known as a bridge or crossover. In contrast, experts may also employ non-analytic strategies like holistic processing to enable rapid, automatic recognition of familiar patterns without detailed analysis (Busey & Vanderkolk, 2005; Chin et al., 2018; Curby et al., 2009; Tanaka & Sengco, 1997). Domains involving perceptual expertise like fingerprint examination often involve both processing approaches—holistic pattern recognition as well as focused analytic attention to minutiae (Busey & Vanderkolk, 2005; Chin et al., 2018). By flexibly shifting between systematic analysis and automatic holistic processing as needed, experts can build rich, organised knowledge structures incorporating both overarching categories and specific item details. This supports accurate and efficient processing and recall of domain-relevant information.

## Experiment 1

Over their careers, fingerprint examiners develop intimate familiarity with the feature relationships that matter when comparing prints. Their increased sensitivity to meaningful patterns, gained through experience, may enhance memory capacity for fingerprints overall (Ericsson & Kintsch, 1995; Gobet & Simon, 1996a, 1996b). This expanded capacity could play a crucial role during comparisons, especially when examiners must sort through banks of potential matches to select candidates for further analysis. Enhanced short-term memory may be particularly beneficial during this selection phase, enabling rapid yet accurate comparison of complex patterns across multiple impressions held briefly in memory. A significant aspect of their job involves this initial selection from many possible matches. Thus, this study aimed to demonstrate whether fingerprint examiners possess superior short-term memory for fingerprints compared to novices, given examiners’ expertise discerning meaningful patterns.

Prior work has provided initial evidence that fingerprint examiners possess superior short-term memory capacity for prints compared to novices (Busey & Vanderkolk, 2005; Thompson & Tangen, 2014). Thompson and Tangen (2014) presented experts and novices with a “crime scene” print briefly (5 s), followed by a 5-s interval and then a second print. Participants had to judge if the prints matched or not. On target trials where prints matched, experts and novices were equally accurate. But on distractor trials with similar non-matching prints, experts were more accurate than novices. By preventing verbal encoding during initial viewing, this paradigm taps into visual short-term memory. The expert advantage for similar non-matches suggests enhanced memory capacity and pattern discrimination ability.

While the prior research provides preliminary indication of fingerprint examiners’ superior short-term memory capacity, its primary purpose was to explore expert performance under limited information conditions. By restricting the amount of information available to participants and preventing verbal encoding, the study illuminates the potential role of non-analytic processes, such as holistic processing, in expert memory (Thompson & Tangen, 2014). However, non-analytic processing alone is likely insufficient to fully support optimal fingerprint comparison performance. For instance, experts generally exhibit superior performance on side-by-side print matching tasks when permitted longer viewing durations, suggesting that slow, analytic-based processing strategies also play a crucial role in fingerprint analysis (Thompson & Tangen, 2014). It is probable that multiple, interacting processing types underlie the exceptional short-term memory capacity demonstrated by experts. In practice, examiners may employ strategies including verbal descriptions or subvocalisation to bolster memory. Constraining verbal encoding could preclude experts from fully employing the range of analytical techniques they have developed through their extensive domain experience. Consequently, such limitations may not capture of the true extent of experts’ memory capabilities shaped through expertise.

Designing human performance studies requires balancing fidelity, generalisability, and control to effectively address the research question (Thompson et al., 2013). The current investigation aims to understand real-world cognitive demands on fingerprint examiners. In a preregistered experiment, we test short-term memory under conditions resembling examiners’ typical tasks. We use a delayed match-to-sample paradigm with sequential presentation of multiple matching candidates to simulate memory processes required in practice. Unlike prior work, we do not limit verbal encoding, enabling natural cognitive strategies. If superior memory partly explains examiners’ accuracy, improving such memory is theoretically and practically important. In sum, by aligning conditions with real-world tasks while permitting normal encoding strategies, this research can provide further insight into the exceptional memory underpinning expert fingerprint analysis.

## Method

### Open practices statement

The methods and materials for Experiment 1 are available on the Open Science Framework, including our experiment code, video instructions, trial sequences, de-identified data, and analysis scripts (https://osf.io/qy2su/).

### Participants

We aimed to recruit as many expert examiners as possible, determining a minimum of 30 to provide sufficient sensitivity (> 0.8) for detecting medium-sized effects. Ultimately, we tested 44 qualified practicing fingerprint experts (25 females; mean age = 43.64 years, SD = 8.41; mean experience = 14.89 years, SD = 7.75) from the Australian Federal Police, Victoria Police, and New South Wales Police. We also tested 44 novices matched on age, gender, and education (25 females; mean age = 43.64 years, SD = 8.67) with no formal fingerprint experience. Novices were recruited from The University of Queensland, The University of Adelaide, and Murdoch University communities, as well as the general public, and compensated AUD$20 per hour. In a single testing session, each participant completed this experiment along with other randomised tasks assessing additional research questions beyond this paper’s scope.^1^

### Materials

The fingerprints used were latent (“crime scene”) and rolled (“arrest”) prints from the Forensic Informatics Biometric Repository (Tear et al., 2010). These high-quality prints with known ground truth were collected from undergraduate students. Rolled exemplars were captured using ink onto standard 10-print cards, fully rolling each finger from nail-edge to nail-edge. Latent prints were lifted from common crime scene surfaces (identified through examiner consultation) including gloss-painted timber, smooth metal, glass, and plastic. To approximate real crime scene variation, participants made contact by actions like “pushing on timber to open a door” or “safely grabbing a knife blade”. Interacting with objects in this way generated realistic latent prints. In summary, the fingerprint stimuli comprised forensically relevant latent and rolled prints collected under controlled conditions from student volunteers.

### Procedure

**Fig. 1:**
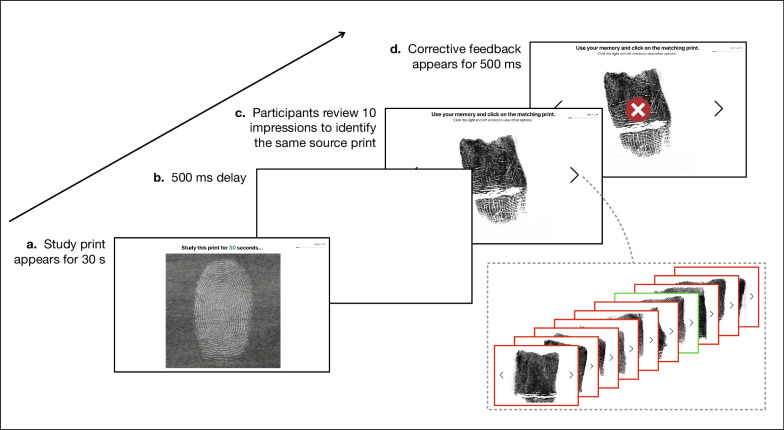
A schematic diagram illustrating the delayed match-to-sample memory task in Experiment 1. *Note.* Each trial begins with a 30-s study phase of a latent fingerprint impression (**a**). After the study phase, there is a 500-ms delay (**b**). Next, a sequence of 10 fully rolled fingerprint impressions is presented (**c**). One of these is the target stimulus, while the remaining nine are distractor stimuli. Following their selection, participants receive corrective feedback for 500 ms before proceeding to the next trial (**d)**

In this experiment, each of the 44 fingerprint experts completed a delayed match-to-sample memory task consisting of 24 trials randomly sampled from a larger pool for each participant (see Fig. 1). On every trial, the experts were first presented with a latent fingerprint impression for 30 s to study. After this 30-s duration elapsed, the latent print disappeared, and there was a 500-ms delay. Next, 10 fully rolled fingerprint impressions subsequently appeared on the screen in sequence. One of these 10 rolled impressions was the same source print, serving as the target stimulus. It corresponded to the same finger as the previously studied latent print, though it was a different, unique impression. The remaining 9 rolled impressions were drawn from different fingers of the same individual who provided the latent print, serving as distractor stimuli. The experts were instructed to carefully sort through the series of rolled impressions as they appeared and select the target print. They had the ability to navigate back and forth between the rolled fingerprint impressions. This navigation was facilitated by on-screen arrows, which could be clicked using a mouse, or by using the arrow keys on the keyboard. To make their selection, participants simply clicked on their chosen fingerprint impression using the mouse. This method allowed them to review the impressions as needed before making a decision. The order of the target and distractor items was randomly shuffled on each trial and the set of 10 rolls remained visible on the screen until the participant made their selection. However, if experts took longer than 20 s to respond on a given trial, a text prompt would appear during the inter-trial interval with the message “Try to decide in less than 20 s”. Once the experts made their selection, brief corrective feedback appeared on the screen for 500 ms before the next trial began. After testing all 44 fingerprint experts on this task, the 44 novice participants subsequently completed the identical set of 24 trial sequences as their matched expert.

### Hypotheses

We hypothesised that novices would perform above chance levels, with a medium effect size (*d* = 0.2 to 0.5). We also predicted experts would perform above chance, but with a large effect size (*d* > 0.5). Thus, when comparing the two groups, we expected experts to outperform novices with a large effect size (*d* > 0.5).

## Results

### Accuracy

**Fig. 2:**
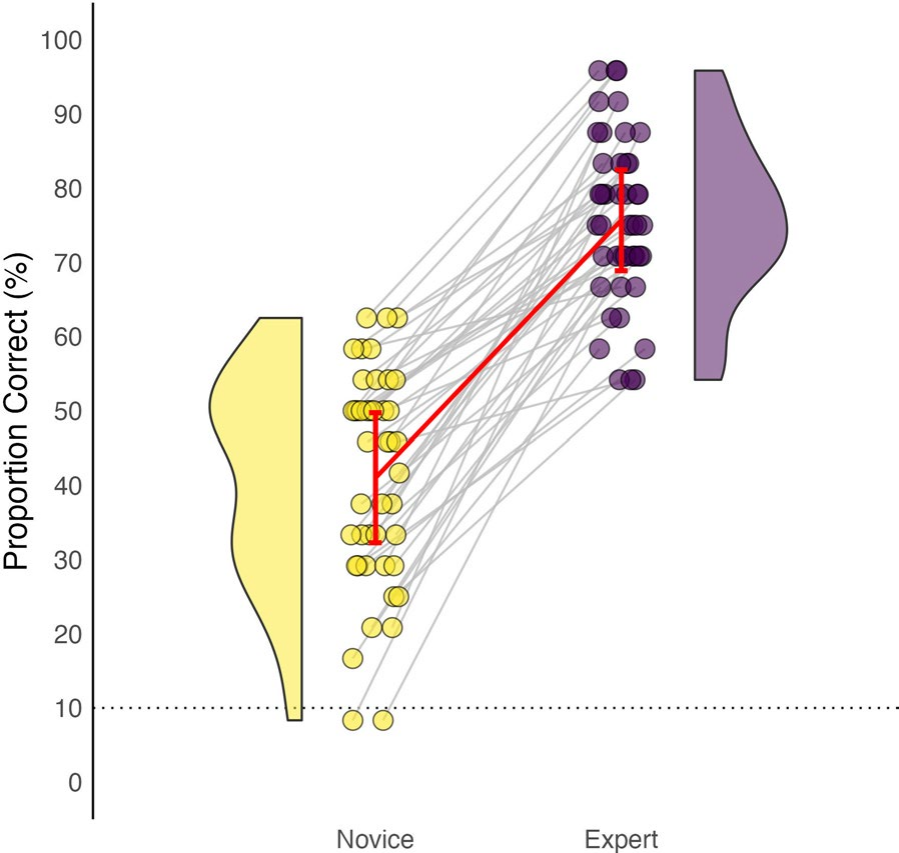
Comparison of proportion correct scores between experts and novices on domain-specific short-term memory tasks. *Note.* Each data point represents the performance of one participant, with experts in purple and novices in yellow. The violin plot shows the distribution of scores for each group, with a wider area indicating a higher density of scores. The dotted line indicates chance performance (10%), and the error bars represent the standard deviation. Each line connects two data points that belong to the same expert-novice pair, who completed the identical set of 24 trial sequences

In Experiment 1, we set out to determine the relative performance of experts and novices on our test of short-term memory. The individual performance of each participant is represented in Fig. 2. First, we performed one-sample *z* tests^2^ for proportions, comparing the mean proportion correct scores of experts and novices to chance level performance (10%). Our results show that both experts (M = 76%, SD = 11%) and novices (M = 41%, SD = 14%) performed significantly above chance, as indicated by the dotted line in Fig. 2, *z* = 14.52, *p* < 0.001, *h* = 1.47, 95% CI [62.98%, 88.34%], *z* = 6.86, *p* < 0.001, *h* = 0.75, 95% CI [26.47%, 55.54%], respectively. A two-sample *z* test for differences in proportions revealed that the difference between the two groups was significant, suggesting that experts outperform novices on this task, *z* = 3.30, *p* < 0.001, *h* = 0.72, 95% CI [14.06%, 55.26%].

### Exploratory analyses

#### Speed–accuracy

We conducted exploratory analyses examining the relationship between response speed and accuracy to determine if experts’ superior performance could be explained by differing speed–accuracy trade-offs between groups. Correlation analysis revealed response time positively correlated with accuracy for novices, *r*(42) = 0.49, *p* < 0.001, but not experts, *r*(42) = 0.07, *p* = 0.646. While no speed–accuracy trade-off was evident for experts, novices’ accuracy appeared to be related to response speed. To better account for these observed speed–accuracy differences, we computed Balanced Integration Scores (BIS) combining both measures (Liesefeld & Janczyk, 2019; Vandierendonck, 2018). BIS is a measure designed to equally consider response time and accuracy. It is determined by first standardising both response time and accuracy scores for correct responses only, as incorrect responses tend to be more influenced by speed–accuracy trade-offs. Each standardised accuracy score is then subtracted from the standardised response time score, yielding a composite speed–accuracy score for every participant. BIS is a reliable measure for assessing speed–accuracy balance, as it minimises the impact of individual differences in speed–accuracy trade-offs (Liesefeld & Janczyk, 2019). The average BIS is zero with a standard deviation of one. Scores above zero signify performance that is better than the mean, while scores below zero indicate performance that is lower than the mean. Between-groups *t* test on BIS showed experts still significantly outperformed novices when accounting for response time, *t*(86) = 8.23, *p* < 0.001, *d* = 1.76.

#### Correlating performance and experience

We also conducted correlation analysis between years of fingerprint identification experience and performance on the memory task. For experts, no significant correlation emerged between years of experience and accuracy, *r*(42) = 0.01, *p* = 0.972, or between experience and speed–accuracy performance, *r*(42) = 0.05, *p* = 0.764.

## Discussion

In Experiment 1, we compared expert fingerprint examiners and inexperienced novices on a short-term memory task requiring fingerprint identification. Results revealed both groups performed significantly above chance, confirming the task’s efficacy. However, as hypothesised, experts demonstrated substantially superior performance recognising images from their domain of expertise compared to novices. These findings align with existing research indicating domain-specific expertise, like fingerprint examination, can substantially enhance memory capabilities (see Ericsson, 2018 for a review). In sum, experts showed exceptional short-term retention for fingerprints versus novices, highlighting how specialised experience refines memory skills within one’s field.

Like many other areas of expertise, fingerprint examiners do not explicitly train their memory for fingerprint configurations. However, we suspect that many of the tasks performed by fingerprint examiners rely heavily on short-term memory. An examiner analyses the ridge patterns of a crime scene print, noting distinguishing configurations and their positions, and then sorts through a database of suspect prints until they find one suitable for more thorough comparison (Ashbaugh, 1999; Daluz, 2018; Ulery et al., 2011). Short-term memory appears crucial for examiners, especially throughout this selection phase. They may use their memory to accurately reflect the latent print’s features, allowing for rapid yet flexible investigation of a large number of potential matching impressions. While not directly trained, robust short-term memory seems vital for fingerprint analysis, enabling examiners to efficiently search prints by retaining key details of the latent.

One likely possibility is that experts develop more effective organisational processing strategies, allowing efficient recognition of familiar patterns in their domain (Ericsson & Kintsch, 1995; Gobet & Simon, 1996a, 1996b). With careers spanning decades, examiners gain sensitivity to meaningful patterns unavailable to novices. An examiner’s increased sensitivity likely enables integrating many fingerprint features and positions into one representation. By consolidating information into a coherent mental template, experts can better manage short-term memory’s limited resources, contributing to their superior performance (Ericsson & Kintsch, 1995; Gobet & Simon, 1996a, 1996b). In essence, experts may excel by forming consolidated templates that efficiently capture fingerprint details, overcoming limitations in short-term retention.

## Experiment 2

In Experiment 1, we demonstrated experts outperformed novices on a short-term memory test involving domain-specific items. This supports expertise enhancing organisational processing, enabling more effective encoding and retrieval within one’s specialty (Ericsson & Kintsch, 1995; Gobet & Simon, 1996a, 1996b). In Experiment 2, we explore conditions influencing performance differences between experts and novices.

An item’s memorability depends on unique processing and distinction from other stored items (Hunt, 2006; Konkle et al., 2010; Rawson & Van Overschelde, 2008). For example, distinctive or striking images like a dog holding a smoking pipe in its mouth or an airplane crash are better remembered when embedded among mundane scenes, potentially due to greater attention during encoding (Hunt, 2003; Standing, 1973). Memory models generally agree that interference, or the competition between similar memories, makes it harder to recall specific items. However, when an item has unique features, it is easier to retrieve because its distinctiveness reduces interference (Shiffrin & Steyvers, 1997).

There have been few studies exploring the role of distinctiveness and memory in the context of perceptual expertise. For example, radiologists demonstrated better memory overall compared to novices and also showed better performance for mammograms depicting medical anomalies compared to those featuring normal cases (Schill et al., 2021). Abnormal images contain distinct features apparent to experts, making them more memorable compared to other items; conversely, novices fail to appreciate these attributes, causing the abnormal images to be indistinct from others in memory. Novice participants showed slightly improved memory performance for normal versus abnormal images, potentially attributable to greater dissimilarity of normal cases in the set, rendering them more visually distinctive. Perceptual expertise allows recognition of distinct attributes enhancing memorability, while novices’ lack of expertise diminishes distinctiveness between abnormal and normal cases.

The influence of distinctiveness on memory is particularly relevant to fingerprint analysis, where experts discern differences among numerous prints. Relevant features experts rely on may be perceptually distinctive ridge flow variations. During think-aloud tasks where examiners verbalise thoughts deciding print matches, they often focus on features perceptually grabbing attention, describing searching for ridges “popping out”, “standing out”, or “sticking out”, especially in challenging cases (Corbett & Tangen, 2023). Perceptual distinctiveness likely aids identifying critical fingerprint differences and may enhance fingerprint memory. While lacking domain knowledge, novices may still benefit from item distinctiveness. Perceptual distinctiveness appears to direct experts’ focus during comparison to key differentiating fingerprint features, potentially improving memory. Novices without domain expertise may also show better memory for more distinctive prints.

In Experiment 2, we are particularly interested in understanding the conditions under which experts exhibit the most significant performance gap in memory compared to novices. Using the same memory task as in Experiment 1, we incorporate both distinctive and nondistinctive fingerprints, allowing us to examine the interplay between expertise and distinctiveness. In essence, the visually distinctive prints might serve as a cognitive anchor for novices, allowing them to better remember and recognise these items compared to prints with less distinctive features. This benefit, however, may not be as pronounced for experts, who possess the specialised knowledge and skills to discern subtle differences among both distinctive and nondistinctive prints. In summary, we predict distinctive prints will improve novices’ memory performance by providing cognitive anchors, while experts’ specialised perceptual skills will minimise differences between memorising distinctive versus nondistinctive prints.

## Methods

### Open practices statement

The methods and materials for Experiment 2 are available on the Open Science Framework, including our experiment code, video instructions, trial sequences, de-identified data, and analysis scripts (https://osf.io/x6caz/).

### Participants

Based on the large effect size found in Experiment 1, we determined that 30 participants per group would provide sufficient sensitivity (power = 1.0) to detect a medium effect size. We tested 30 qualified practicing fingerprint experts (6 females and 24 males, mean age = 45.73 years, SD = 6.44, mean experience = 10.80, SD = 5.38) from Victoria Police, Western Australia Police and the Queensland Police Service. We also tested 30 novices (24 females and 6 males, mean age = 21.47 years, SD = 7.77) who had no formal experience with fingerprints. Novices were recruited from The University of Queensland and were awarded course credit for their participation.

### Materials

We sourced our fingerprints from the NIST Special Database 300 (Fiumara et al., 2018). The collection comprised both plain and fully rolled impressions (often termed “arrest” prints) that were originally obtained in real-world policing settings, ensuring a representative variability in print quality. For our analysis, we selected 200 individual plain impressions, each paired with 10 corresponding fully rolled prints, totalling 2000 prints.

#### Print distinctiveness

To gather fingerprints that were perceptually distinctive or nondistinctive, we had a separate group of 25 fingerprint experts and 25 novices without experience rate a database of 6000 fingerprints on a scale of distinctiveness. The distinctiveness of each fingerprint was rated using a Likert scale, with options ranging from 1 (not distinctive) to 7 (highly distinctive). “Distinctiveness” was broadly defined for both experts and novices as the degree to which a fingerprint stands out in a crowd of other fingerprints for any reason. This open-ended definition allowed raters the flexibility to consider any aspect of the fingerprint that they felt made it more or less noticeable or memorable, without restricting them to specific features like ridge patterns or minutiae.

Ratings from both expert and novice groups were first compiled and averaged for each fingerprint, producing separate mean distinctiveness scores from each cohort. To address potential variations in rating scales and tendencies between the groups, these scores were then standardised using z-scores, which facilitated a direct comparison by quantifying how many standard deviations a fingerprint’s average rating was from the mean rating of each respective group. The z-scores from both groups for each fingerprint were combined by calculating their mean, ensuring that the final score represented a consensus across both expert and novice evaluations. This process culminated in the selection of the 100 fingerprints with the highest and lowest mean z-scores, denoting the most and least distinctive prints, respectively. Importantly, we ensured there were no duplicate prints selected between or within the two conditions of most and least distinctive.

We combined the ratings from both experts and novices, rather than relying solely on expert judgments, because we did not want to inadvertently select only fingerprints containing features that catered to expert-level perceptions of distinctiveness. By also incorporating novice ratings, we aimed to select a sample of distinctive and nondistinctive prints that reflected a consensus across both levels of experience. This approach allows us to better understand how both experts and novices perceive and process the same set of fingerprints.

### Procedure

We used the same delayed match-to-sample memory task as in Experiment 1, with the exception of print study time, and the number of trials. Further piloting of this experiment revealed that experts and novices tended to perform similarly with a 10-s study time as they did with 30 s. In order to save time, we decided to give participants only 10 s to study each impression. There were 36 trials in this experiment. Based on the large expert-novice difference found in Experiment 1, we anticipated a large difference in performance between professional fingerprint examiners and novices. As such, we calculated that 36 observations (18 per condition: distinctive, nondistinctive) from 30 participants per group would provide sufficient sensitivity (power = 1.0) to detect a medium effect size.

### Hypotheses

Expert fingerprint examiners should have better overall memory performance than inexperienced novices, as they possess domain-specific knowledge and experience that allow them to process and organise fingerprint information more efficiently. Both experts and novices should perform better on the distinctive fingerprint condition compared to the nondistinctive condition. Distinctive fingerprints are more likely to stand out, making them easier to remember and recognise for both groups.

The difference in memory performance between experts and novices might be greater in the nondistinctive condition compared to the distinctive condition. This is because experts, with their specialised knowledge, may be better equipped to discern subtle differences among nondistinctive fingerprints than novices, thus reducing the impact of low distinctiveness for experts. In the distinctive condition, the advantage of the experts may be less pronounced, as both groups benefit from the distinctiveness of the stimuli, potentially reducing the performance gap between them.

## Results

### Accuracy

**Fig. 3:**
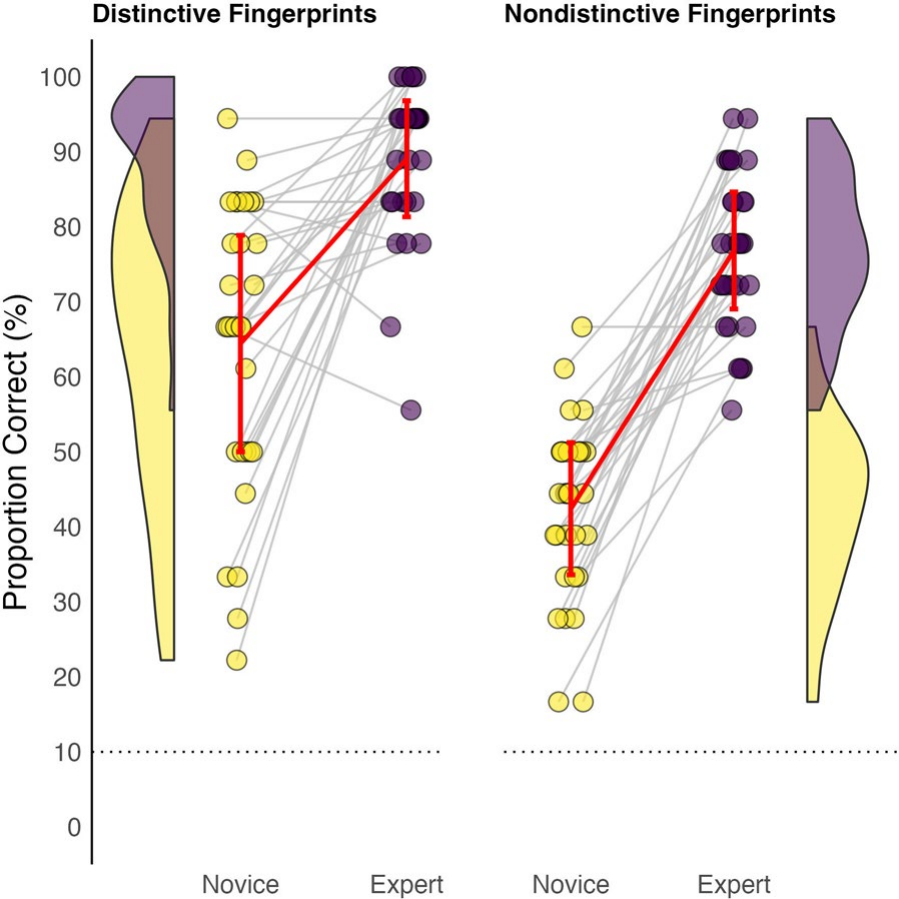
Comparison of proportion correct scores between experts and novices on domain-specific short-term memory tasks for distinctive and nondistinctive fingerprints. *Note.* Each data point represents the performance of one participant, with experts in purple and novices in yellow. The error bars represent the standard deviation. The dotted line indicates chance performance (10%). Each line connects two data points that belong to the same expert-novice pair, who completed the identical set of 24 trial sequences

The performance of each expert and novice given the distinctive and nondistinctive prints is represented in Fig. 3. Our analysis strategy was guided by the nature of our accuracy data,^3^ which exhibited characteristics unsuitable for traditional linear analysis. Assumption tests on accuracy data revealed that our data were not normally distributed. Specifically, we found that experts’ performance on distinctive items was negatively skewed. We also found heterogeneous variance in the performance of expert and novice groups for the distinctive items. To address these challenges, we employed a generalised linear model (GLM) with a binomial distribution. This choice was driven by the binary and bounded nature of our outcome variable (correct or incorrect responses) and the model’s robustness in handling data with non-normal distributions and varying variances. The GLM approach is particularly adept at analysing binary outcomes, allowing us to investigate the effects of expertise level and stimulus type on accuracy in a manner that is both statistically appropriate and insightful for our research questions.

A GLM with a binomial distribution was conducted to investigate the effects of Expertise (expert, novice) and stimulus type (distinctive, nondistinctive), as well as their interaction, on the accuracy of fingerprint identification. The analysis revealed significant main effects for both group and stimulus type. Specifically, being in the novice group was associated with a decrease in the log odds of correct identification compared to experts (*B* =  − 1.50, SE = 0.16, *z* =  − 9.13, *p* < 0.001). Similarly, the nondistinctive stimulus type was associated with a decrease in the log odds of correct identification compared to distinctive stimuli (*B* =  − 0.90, SE = 0.17, *z* =  − 5.24, *p* < 0.001). Contrary to our predictions, the interaction between group and stimulus type was not statistically significant (*B* =  − 0.00, SE = 0.21, *z* =  − 0.01, *p* = 0.991), suggesting that the difference in performance between experts and novices was similar across both distinctive and nondistinctive stimuli. The model fit was indicated by a reduction in deviance from the null model (Residual deviance = 2395.9 on 2156 degrees of freedom; Null deviance = 2701.7 on 2159 degrees of freedom).

### Exploratory analyses

#### Speed–accuracy

We also explored whether fingerprint experts are more accurate than novices, or whether their superior performance can be explained by differences in speed–accuracy trade-offs across the two groups. When examining the correlation between response time and accuracy, we did not find a significant correlation for either group: experts *r*(42) = −0.04, *p* = 0.829, and novices *r*(42) = −0.35, *p* = 0.056. To further examine whether differences in speed–accuracy account for the differences in accuracy, we first computed each participant’s Balanced Integration Score. Like our accuracy data, we found that Balanced Integration Scores were also negatively skewed in some cells and some to have heterogeneous variances between groups. We conducted both parametric and the equivalent non-parametric tests given the violations of normality and homogeneity of variance. We found the pattern of effects across both models so we only report results from the analysis of variance (see https://osf.io/x6caz/ for all analyses).

We subjected the Balanced Integration Scores to a 2 (Expertise: expert, novice) × 2 (Stimulus Type: distinctive, nondistinctive) mixed analysis of variance. The results revealed that when taking into account response time, the main effect of Expertise and Stimulus Type still remain, *F*(1, 58) = 26.39, *p* < 0.001, *η*^2^_*G*_ = 0.25 and *F*(1, 58) = 93.27, *p* < 0.001, *η*^2^_*G*_ = 0.31, respectively. That is, experts still outperform novices overall, and participants perform better on distinctive trials compared to nondistinctive trials. However, we did not find the expected interaction, *F*(1, 58) = 0.62, *p* = 0.434, η^2^_G_ =  < 0.01.

#### The relationship between performance and experience

When correlating years of experience in fingerprint identification with performance on the memory task, we find no significant associations. For fingerprint experts, there was no correlation between their years of experience and their accuracy,* r*(28) = 0.32, *p* = 0.086, or between years of experience and speed–accuracy, *r*(28) = 0.03, *p* = 0.882.

## Discussion

In Experiment 2, we aimed to explore conditions influencing performance differences between expert fingerprint examiners and novices on a fingerprint short-term memory task. Specifically, we examined the impact of print distinctiveness on memory and its contribution to the expertise advantage. Replicating Experiment 1, results showed both groups performed above chance, with experts demonstrating substantially higher performance recognising domain-relevant images. This difference likely stems from experts’ specialised knowledge and experience enabling more efficient fingerprint information processing and organisation (Ericsson & Kintsch, 1995; Gobet & Simon, 1996a, 1996b). In essence, experts outweighed novices in memory for fingerprints, aligning with their perceptual expertise.

In line with our predictions, we also found that both experts and novices performed better in the distinctive fingerprint condition compared to the nondistinctive condition, supporting our hypothesis that distinctive prints are more memorable for both groups (Hunt, 2006; Konkle et al., 2010; Rawson & Van Overschelde, 2008). However, experts still outperformed novices even for distinctive prints, suggesting that expertise remains a major factor in memory performance.

We predicted an interaction effect, where the expert-novice performance gap would be more pronounced for nondistinctive prints. We reasoned that expertise would play a vital role in discerning subtler differences among less distinctive prints, as experts’ specialised knowledge and experience might better equip them to distinguish these differences, thereby reducing the impact of low distinctiveness (Schill et al., 2021). However, our analysis revealed a consistent difference in performance between experts and novices that persisted regardless of print distinctiveness. This finding suggests that while distinctiveness itself remains a factor in overall performance, the impact of fingerprint expertise on memory is a robust phenomenon, not markedly influenced by the distinctiveness of the prints.

## General discussion

People with expertise often exhibit remarkable memory capabilities (Ericsson, 2018). Our investigation show that fingerprint examiners are no exception. Across two experiments, experts demonstrated superior memory for domain-relevant images compared to novices. In Experiment 2, we showed distinctiveness also plays a role in supporting memory. Both experts and novices performed better with distinctive versus nondistinctive prints, and our analysis indicated that the performance gap between experts and novices was consistent across both types of stimuli.

Our results from both experiments showed no significant associations between years of experience and performance on the memory tasks for fingerprint experts. This suggests that the tasks might not have been sensitive enough to differentiate between varying experience levels, or that years of experience is not an accurate measure of expertise. The latter point is reflected in studies across similar fields. For instance, White et al. (2015a) found no significant correlation between the length of employment and the accuracy of face examiners, indicating that the number of years in a profession does not necessarily reflect true skill. Further research shows that expertise in certain fields tends to plateau relatively early in one’s career (Choudhry et al., 2005; Ericsson, 2004). Future studies should employ more valid job performance assessments (Shanteau et al., 2002) to offer a more comprehensive evaluation of their level of expertise and its influence on task performance.

The results have meaningful implications for training and assessment in fingerprint analysis, as the short-term memory differences between experts and novices shed light on cognitive processes underlying expertise. Memory appears crucial, enabling experts to retain critical details and efficiently compare prints. By enhancing memory performance, examiners could potentially improve their ability to recognise subtle differences and minimise the likelihood of errors in fingerprint identification. Such enhancement might involve targeted training exercises focusing on retention and recall of complex fingerprint features under varying conditions, closely mirroring the challenges encountered in real-world scenarios. However, this presupposes that the same cognitive mechanisms at work in expert fingerprint examination are also at work in our memory task. If experts do not use similar processes across these tasks, then boosting their memory for prints may have little impact on their overall performance. The focus then shifts from whether there is a difference between experts and novices to how a more effective short-term memory supports better performance. Longitudinal studies will be necessary to describe the development of expert performance and how mechanisms that mediate short-term memory are first established and then developed to meet the changing memory demands as an expert improves.

Our exploration of how distinctiveness and memory interact is also of theoretical and practical interest. Fingerprint examination involves identifying features within prints to compare and match them. Our findings suggest that experts may rely on short-term memory for fast, accurate critical decisions and the visual distinctiveness of prints may aid this process for both experts and novices. Given memory’s apparent role, it seems important to consider how we can leverage the distinctiveness of prints in memory.

The process of categorising and comparing fingerprint features presents significant perceptual challenges, especially with low-quality or distorted prints. Experts must become accustomed to the many potential variations in prints and identify the most individualising features. Understanding how factors like pressure, movement, recording method, humidity, and surface alter fingerprint deposition allows examiners to better discriminate relevant from irrelevant distinguishing information. For instance, some visually distinctive features may not contribute to identification, potentially distracting novices. Conversely, other visually distinctive features, such as unique minutiae combinations or unusual ridge flow patterns, are critical for accurate identification. Understanding the most relevant distinctive features is an important step in developing foundational skills for accurate fingerprint identification. For example, a novice might learn that certain ridge distortions, while noticeable, are not intrinsic to the fingerprint pattern but are caused by the pressure of finger placement. By acquiring this knowledge, they can enhance their ability to discern between important and less important visual cues, leading to improved performance in recognising fingerprints.

There may be other ways to leverage distinctiveness effects on memory. Our manipulation likely used prints perceptually distinctive to both groups. However, feature rarity likely also contributes to fingerprint distinctiveness. While thinking aloud, examiners noted domain knowledge about commonality of specific minutiae, identifying features as “unique”, “rare”, or “highly common” (Corbett & Tangen, 2023). Assessments of feature rarity may be crucial for accurate judgments, since rarer features can serve as diagnostic cues for discrimination and categorisation (Busey et al., 2017). Prints sharing a rare feature (e.g. a “trifurcation”) would be more likely to match than those sharing a common feature (e.g. a “bifurcation”; Gutiérrez-Redomero et al., 2012). Evidence suggests experts may be attuned to such statistical information. In one study, fingerprint examiners were better able to discriminate between rare and common broad fingerprint patterns (e.g. tented arch vs. left plain loop) than novices (Growns et al., 2023).

While the true statistical rarity of fingerprint patterns and minutiae remains unknown, experts likely develop intuitive statistical knowledge from extensive casework, allowing efficient discernment and processing of critical patterns and minutiae. These features may not be perceptually unique; however, understanding rarity could leverage conceptual distinctiveness to improve memory for these items. This idea is supported by Schill et al. (2021); radiologists remembered mammograms with abnormalities (anomalies) better than normal ones, whereas novices showed slightly better memory for normal cases. Abnormal images contain distinct features apparent to experts, enhancing memorability over other items, while novices fail to appreciate these attributes. Normal images were more visually distinctive, benefiting novice memory. This highlights the importance of conceptual distinctiveness knowledge for memory in perceptual expertise.

As with radiologists and mammogram abnormalities, fingerprint examiners develop sensitivity to conceptually varying print features through experience, allowing more accurate comparative judgments. This knowledge likely aids their memory performance by drawing attention to conceptually unique differentiating features between prints. More research on distinctiveness and diagnostic value of various ridge formations would be beneficial, as distinctiveness judgments currently rely on subjective experience. Research on the discriminating value of ridge formations would provide a useful framework for future statistical models and provide courts with more information to consider when evaluating the reliability of the science. Such research could also provide examiners an objective basis for their intuitive knowledge gained through experience, serving as an excellent training tool.

Training programmes could emphasise understanding rarity and uniqueness of certain features, prompting trainees to focus on these factors when evaluating distinctiveness. Indeed, there is evidence showing that this kind of training can improve accuracy on fingerprint comparison tasks. Growns et al. (2022) demonstrated that training individuals to use statistically diagnostic features in fingerprint comparison enhanced the performance of both novices and professional examiners. In a similar field, Towler et al. (2021) found training novices to focus on facial features most diagnostic of identity, specifically the ears and facial marks, improved accuracy in unfamiliar face matching tasks. We reason that memory might be a mechanism that aids these improvements. By reinforcing conceptual understanding of discriminability, training could leverage distinctiveness effects on memory, enabling both experts and novices to better appreciate and remember distinguishing features. This strategy could enhance examiners’ ability to build comprehensive mental representations of prints focused on critical comparative features. Consequently, this could improve their overall recognition abilities and potentially lead to more accurate fingerprint identifications.

Overall, our findings have significant implications for fingerprint identification, especially amid growing pressure from scientific bodies urging more basic forensic science research (National Academy of Sciences, 2009; President’s Council of Advisors on Science & Technology, 2016). After all, high-stakes decisions with serious consequences often rest on these identifications. There is a risk that an innocent person may be wrongfully incarcerated or that a criminal will be released and commit further crimes. Thus, understanding the nature of forensic expertise is essential to maintain high evidentiary standards. Our findings contribute to the developing literature that seeks to explain the cognitive mechanisms potentially underlying fingerprint examiners’ consistently high performance. By advancing foundational understanding of forensic expertise, this work helps address calls for basic research to uphold integrity in fingerprint analysis, where decisions have real-world impacts.

## Data Availability

The data for each novice and expert participant, and the code used to produce our results and plots, are available on the Open Science Framework, with the exception of identifiable demographic information (Experiment 1: https://osf.io/qy2su/?view_only=917b14dc324a49c7a74c34af65b0888a, Experiment 2: https://osf.io/x6caz/?view_only=9f3534a97f3341e6a838a68ec7a06b54). The images, experimental software, event sequences can also be found through these links.

## Footnotes

1 Along with the primary memory task, participants engaged in nine other domain-specific perceptual tasks: Visual search, hand/fingerprint nomination, side-by-side matching, spot the difference, find-the-fragment, fill-in-the-blank, fragment matching, pattern classification, and a think-aloud task. These tasks collectively took approximately 3 h to complete. To mitigate potential fatigue effects, participants were allowed breaks between tasks. Furthermore, the order of these tasks was randomised for each expert/novice pair, ensuring that each matched pair received the tasks in the same sequence. This approach was designed to reduce systematic influences such as fatigue and practice effects on task performance across expert-novice pairs while maintaining comparability within each pair.

2 The statistical analysis approach for Experiment 1 was updated from the initial plan registered in our pre-registration documents. This change, from t tests to z tests for analysing proportion data, was made to better align with the binary nature of our dataset. For a comprehensive overview of all analyses, including those conducted at different stages of the research see https://osf.io/qy2su/.

3 The analytical approach for this study was refined in response to valuable feedback from peer reviewers. For a comprehensive overview of all analyses, including those conducted at different stages of the research, please refer to https://osf.io/x6caz/.

## References

[CR1] Ashbaugh, D. R. (1999). *Quantitative-qualitative friction ridge analysis: An introduction to basic and advanced ridgeology.* CRC Press.

[CR2] Alvarez GA, Cavanagh P (2004). The capacity of visual short-term memory is set both by visual information load and by number of objects. Current Directions in Psychological Science : A Journal of the American Psychological Society.

[CR3] Baddeley A (2000). The episodic buffer: A new component of working memory?. Trends in Cognitive Sciences.

[CR4] Busey T, Nikolov D, Yu C, Emerick B, Vanderkolk J (2017). Characterizing human expertise using computational metrics of feature diagnosticity in a pattern matching task. Cognitive Science.

[CR5] Busey T, Vanderkolk J (2005). Behavioral and electrophysiological evidence for configural processing in fingerprint experts. Vision Research.

[CR6] Campbell, A. (2011). The fingerprint inquiry report. APS Group Scotland. Retrieved from http://citeseerx.ist.psu.edu/viewdoc/download?doi=10.1.1.361.380&rep=rep1&type=pdf

[CR7] Chase WG, Simon HA (1973). Perception in chess. Cognitive Psychology.

[CR8] Chin MD, Evans KK, Wolfe JM, Bowen J, Tanaka JW (2018). Inversion effects in the expert classification of mammograms and faces. Cognitive Research: Principles and Implications.

[CR9] Choudhry NK, Fletcher RH, Soumerai SB (2005). Systematic review: The relationship between clinical experience and quality of health care. Annals of Internal Medicine.

[CR10] Cole, S. A. (2001). *Suspect identities: A history of fingerprinting and criminal identification*. Harvard University Press.

[CR11] Corbett, B. J., & Tangen, J. M. (2023). Capturing fingerprint expertise with protocol analysis. PsyArXiv. https://osf.io/preprints/psyarxiv/fja8c

[CR12] Cowan N (2001). The magical number 4 in short-term memory: A reconsideration of mental storage capacity. Behavioral and Brain Sciences.

[CR13] Curby KM, Glazek K, Gauthier I (2009). A visual short-term memory advantage for objects of expertise. Journal of Experimental Psychology: Human Perception and Performance.

[CR61] Daluz, H. M. (2018). *Fundamentals of fingerprint analysis* (2nd ed.). Taylor and Francis. 10.4324/9781351043205.

[CR14] de Groot, A. D. (1965). Thought and choice in chess. Mouton Publishers.

[CR15] Dror IE, Mnookin JL (2010). The use of technology in human expert domains: Challenges and risks arising from the use of automated fingerprint identification systems in forensic science. Law, Probability and Risk.

[CR16] Ericsson KA (2004). Deliberate practice and the acquisition and maintenance of expert performance in medicine and related domains. Academic Medicine.

[CR17] Ericsson, K. A. (2018). Superior working memory in experts. In *The cambridge handbook of expertise and expert performance* (pp. 696–713). Cambridge University Press. 10.1017/9781316480748.036

[CR18] Ericsson KA, Kintsch W (1995). Long-term working memory. Psychological Review.

[CR19] Fiumara, G., Flanagan, P., Grantham, J., Bandini, B., Ko, K., & Libert, J. (2018). NIST special database 300: Uncompressed plain and rolled images from fingerprint cards (NIST TN 1993; p. NIST TN 1993). National Institute of Standards and Technology. 10.6028/NIST.TN.1993

[CR20] Gobet F, Simon HA (1996). Recall of random and distorted chess positions: Implications for the theory of expertise. Memory & Cognition.

[CR21] Gobet F, Simon HA (1996). Templates in chess memory: A mechanism for recalling several boards. Cognitive Psychology.

[CR22] Growns B, Mattijssen EJAT, Salerno JM, Schweitzer NJ, Cole SA, Martire KA (2023). Finding the perfect match: Fingerprint expertise facilitates statistical learning and visual comparison decision-making. Journal of Experimental Psychology: Applied.

[CR23] Growns B, Towler A, Dunn JD, Salerno JM, Schweitzer NJ, Dror IE (2022). Statistical feature training improves fingerprint-matching accuracy in novices and professional fingerprint examiners. Cognitive Research: Principles and Implications.

[CR24] Gutiérrez-Redomero E, Rivaldería N, Alonso-Rodríguez C, Martín LM, Dipierri JE, Fernández-Peire MA, Morillo R (2012). Are there population differences in minutiae frequencies? A comparative study of two Argentinian population samples and one Spanish sample. Forensic Science International.

[CR25] Hunt R (2003). Two contributions of distinctive processing to accurate memory. Journal of Memory and Language.

[CR60] Hunt, R. R. (2006). The concept of distinctiveness in memory research. In R. R. Hunt & J. B. Worthen (Eds.), *Distinctiveness and memory* (pp. 3–25). Oxford University Press.

[CR26] Konkle T, Brady TF, Alvarez GA, Oliva A (2010). Conceptual distinctiveness supports detailed visual long-term memory for real-world objects. Journal of Experimental Psychology: General.

[CR27] Lehmann, A. C., & Ericsson, K. A. (1993). Sight-reading ability of expert pianists in the context of piano accompanying. *Psychomusicology**: **A Journal of Research in Music Cognition, 12*(2), 182–195. 10.1037/h0094108

[CR28] Lesgold A, Rubinson H, Feltovitch P, Glaser R, Klopher D, Wang Y, Chi MTH, Glaser R, Farr MJ (1988). Expertise in a complex skill: Diagnosing X-ray pictures. The nature of expertise.

[CR29] Liesefeld HR, Janczyk M (2019). Combining speed and accuracy to control for speed-accuracy trade-offs(?). Behavior Research Methods.

[CR30] Luck SJ, Vogel EK (1997). The capacity of visual working memory for features and conjunctions. Nature (london).

[CR31] Meinz EJ, Hambrick DZ (2010). Deliberate practice is necessary but not sufficient to explain individual differences in piano sight-reading skill: The role of working memory capacity. Psychological Science.

[CR32] Miller GA (1956). The magical number seven, plus or minus two: Some limits on our capacity for processing information. Psychological Review.

[CR33] National Academy of Sciences. (2009). Strengthening forensic science in the United States: A path forward. Retrieved from https://www.ncjrs.gov/pdffiles1/nij/grants/228091.pdf

[CR34] Peeck J, Zwarts J (1983). Recognition memory for pictures of birds in relation to bird-watching skill. The American Journal of Psychology.

[CR35] President’s Council of Advisors on Science and Technology. (2016). Forensic science in criminal courts: Ensuring scientific validity of feature-comparison methods. Retrieved from https://obamawhitehouse.archives.gov/sites/default/files/microsites/ostp/PCAST/pcast_forensic_science_report_final.pdf

[CR36] Rawson KA, Van Overschelde JP (2008). How does knowledge promote memory? The distinctiveness theory of skilled memory. Journal of Memory and Language.

[CR37] Searston RA, Tangen JM (2017). Expertise with unfamiliar objects is flexible to changes in task but not changes in class. PLoS ONE.

[CR38] Schmidt HG, Rikers RMJP (2007). How expertise develops in medicine: Knowledge encapsulation and illness script formation. Medical Education.

[CR39] Schill HM, Wolfe JM, Brady TF (2021). Relationships between expertise and distinctiveness: Abnormal medical images lead to enhanced memory performance only in experts. Memory & Cognition.

[CR40] Shanteau J, Weiss DJ, Thomas RP, Pounds JC (2002). Performance-based assessment of expertise: How to decide if someone is an expert or not. European Journal of Operational Research.

[CR41] Shiffrin RM, Steyvers M (1997). A model for recognition memory: REM—retrieving effectively from memory. Psychonomic Bulletin & Review.

[CR42] Standing L (1973). Learning 10,000 pictures. Quarterly Journal of Experimental Psychology.

[CR43] Tear, M. J., Thompson, M.B., & Tangen, J. M. (2010, September 27–October 1) The importance of ground truth: an open source biometric repository [Paper presentation]. *Proceedings of the 54th annual meeting of the Human Factors and Ergonomics Society*, San Francisco, CA, USA. 10.1177/154193121005401923

[CR44] Tangen JM, Thompson MB, McCarthy DJ (2011). Identifying fingerprint expertise. Psychological Science.

[CR45] Tanaka JW, Sengco JA (1997). Features and their configuration in face recognition. Memory & Cognition.

[CR46] Thompson WC, Cole SA (2005). Lessons from the Brandon mayfield case. The Champion.

[CR47] Thompson MB, Tangen JM (2014). The nature of expertise in fingerprint matching: Experts can do a lot with a little. PLoS ONE.

[CR48] Thompson MB, Tangen JM, McCarthy DJ (2013). Expertise in fingerprint identification. Journal of Forensic Sciences.

[CR49] Towler, A., Keshwa, M., Ton, B., Kemp, R. I., & White, D. (2021). Diagnostic feature training improves face matching accuracy. *Journal of Experimental Psychology*: *Learning, Memory, and Cognition, 47*(8), 1288–1298. 10.1037/xlm0000972 10.1037/xlm0000972 33914576

[CR50] Towler A, White D, Kemp RI (2017). Evaluating the feature comparison strategy for forensic face identification. Journal of Experimental Psychology: Applied.

[CR51] Ulery BT, Hicklin RA, Buscaglia J, Roberts MA (2011). Accuracy and reliability of forensic latent fingerprint decisions. Proceedings of the National Academy of Sciences.

[CR52] Vandierendonck A (2018). Further tests of the utility of integrated speed-accuracy measures in task switching. Journal of Cognition.

[CR53] Vogel EK, Woodman GF, Luck SJ (2001). Storage of features, conjunctions, and objects in visual working memory. Journal of Experimental Psychology: Human Perception and Performance.

[CR54] Vogelsang MD, Palmeri TJ, Busey TA (2017). Holistic processing of fingerprints by expert forensic examiners. Cognitive Research: Principles and Implications.

[CR55] Ward P, Williams AM (2003). Perceptual and cognitive skill development in soccer: The multidimensional nature of expert performance. Journal of Sport and Exercise Psychology.

[CR56] Wheeler ME, Treisman AM (2002). Binding in short-term visual memory. Journal of Experimental Psychology. General.

[CR57] White D, Dunn JD, Schmid AC, Kemp RI (2015). Error rates in users of automatic face recognition software. PLoS ONE.

[CR58] White D, Phillips PJ, Hahn CA, Hill M, O’Toole AJ (2015). Perceptual expertise in forensic facial image comparison. Proceedings of the Royal Society b: Biological Sciences.

[CR59] Zhang W, Luck SJ (2008). Discrete fixed-resolution representations in visual working memory. Nature.

